# Supplementary material for: High-Output Lotus-Leaf-Bionic Triboelectric Nanogenerators Based on 2D MXene for Health Monitoring of Human Feet
Source: Nanomaterials (Basel). 2022 Sep 16;12(18):3217. doi: 10.3390/nano12183217 (PMC9500758; doi:10.3390/nano12183217)
Supplement: Supplementary file 1 [file nanomaterials-12-03217-s001.zip › nanomaterials-1822422 - Supplementary Materials.pdf]

nanomaterials

Supporting Information

High-Output Lotus-Leaf-Bionic Triboelectric Nanogenerators Based on 2D MXene for Health Monitoring of Human Feet

Like Wang, Huichen Xu, Fengchang Huang, Xiaoma Tao, Yifang Ouyang, Yulu Zhou * and Xiaoming Mo *

Center on Nanoenergy Research, Guangxi Key Laboratory for Relativistic Astrophysics, School of Physical Science and Technology, Guangxi University, Nanning 530004, China

* Correspondence: ylzhou@gxu.edu.cn (Y.Z.); xmmo@gxu.edu.cn (X.M.)

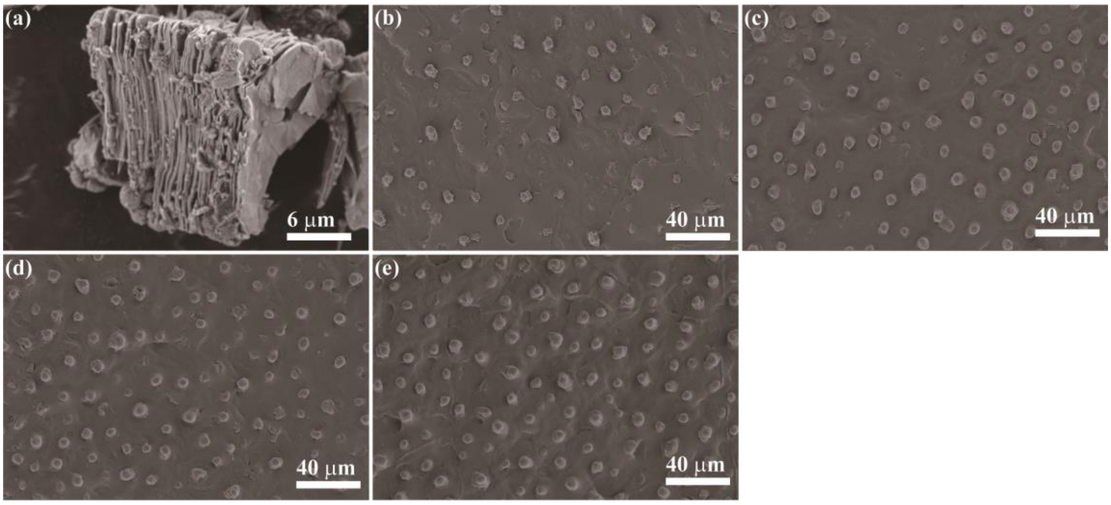

Figure S1. (**a**) Morphology of the MXene material ($Ti_3C_2T_x$) used for doping. (**b–e**) Surface morphologies of the MXene-doped PDMS with different content of MXene: (**b**) 0 wt.%, (**c**) 5 wt.%, (**d**) 10 wt.%, (**e**) 15 wt.%.

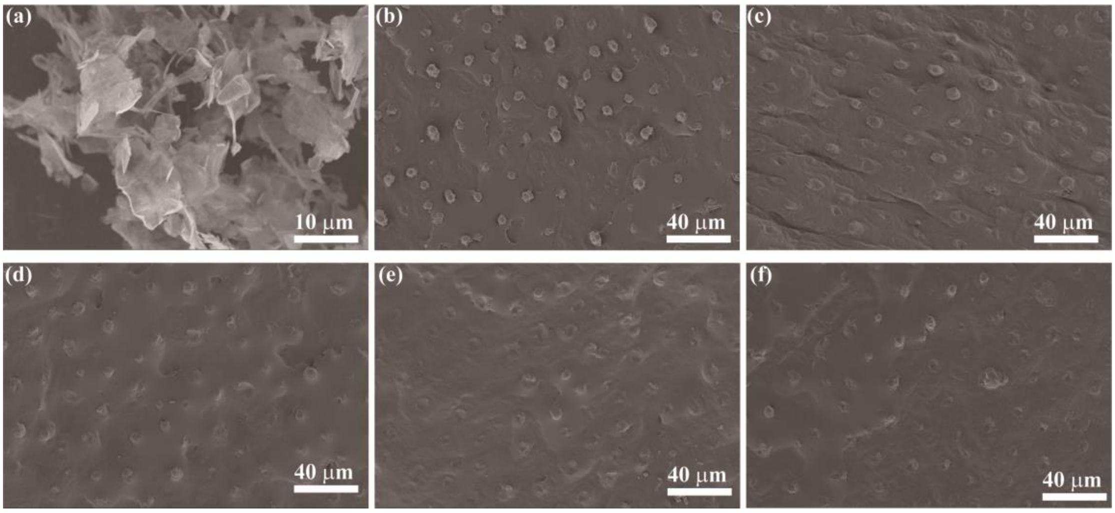

Figure S2. (**a**) SEM image of the Graphene material used for control experiments. (**b–f**) Morphologies of the Graphene-doped PDMS with different content of Graphene: (**b**) 0 wt.%, (**c**) 0.2 wt.%, (**d**) 0.4 wt.%, (**e**) 0.6 wt.%, (**f**) 0.8 wt.%.

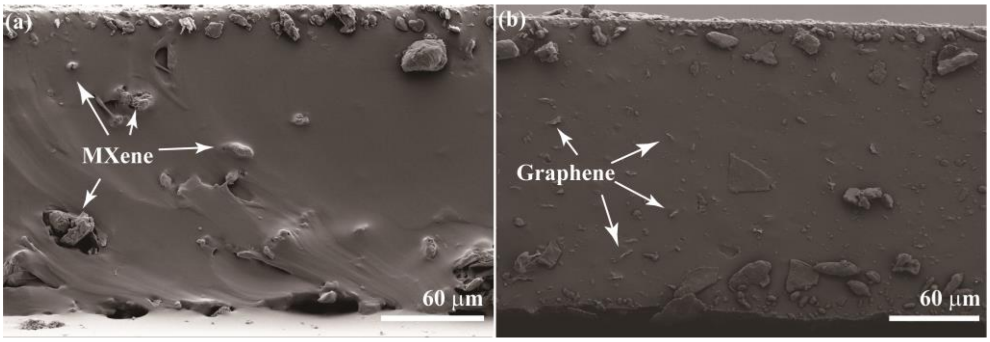

Figure S3. Cross-section SEM images for different negative triboelectric layer: (**a**) MXene-doped PDMS (10 wt.%). (**b**) Graphene-doped PDMS (0.6 wt.%).

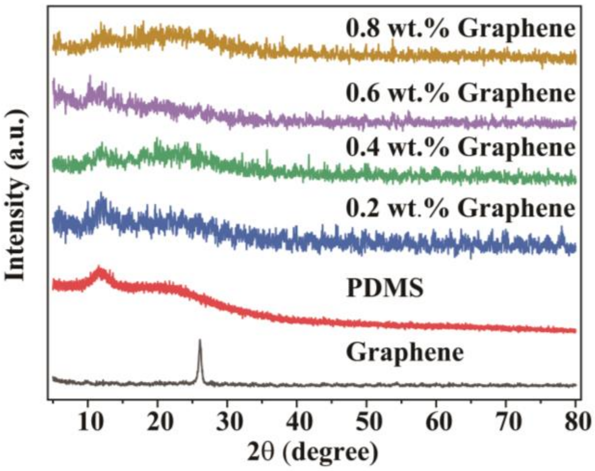

Figure S4. XRD patterns of the Graphene-doped PDMS films in comparison with pure PDMS and Graphene.

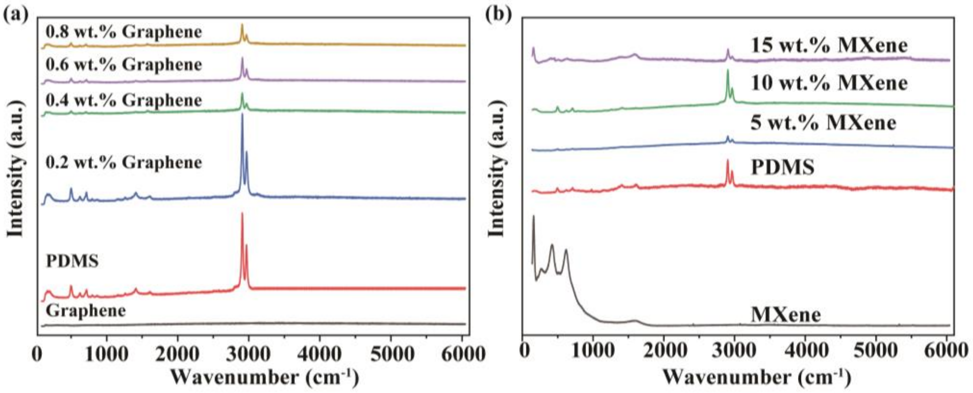

Figure S5. Raman spectra for: (**a**) Graphene-doped PDMS film in comparison with pure PDMS and Graphene, and (**b**) MXene-doped PDMS films in comparison with pure PDMS and MXene.

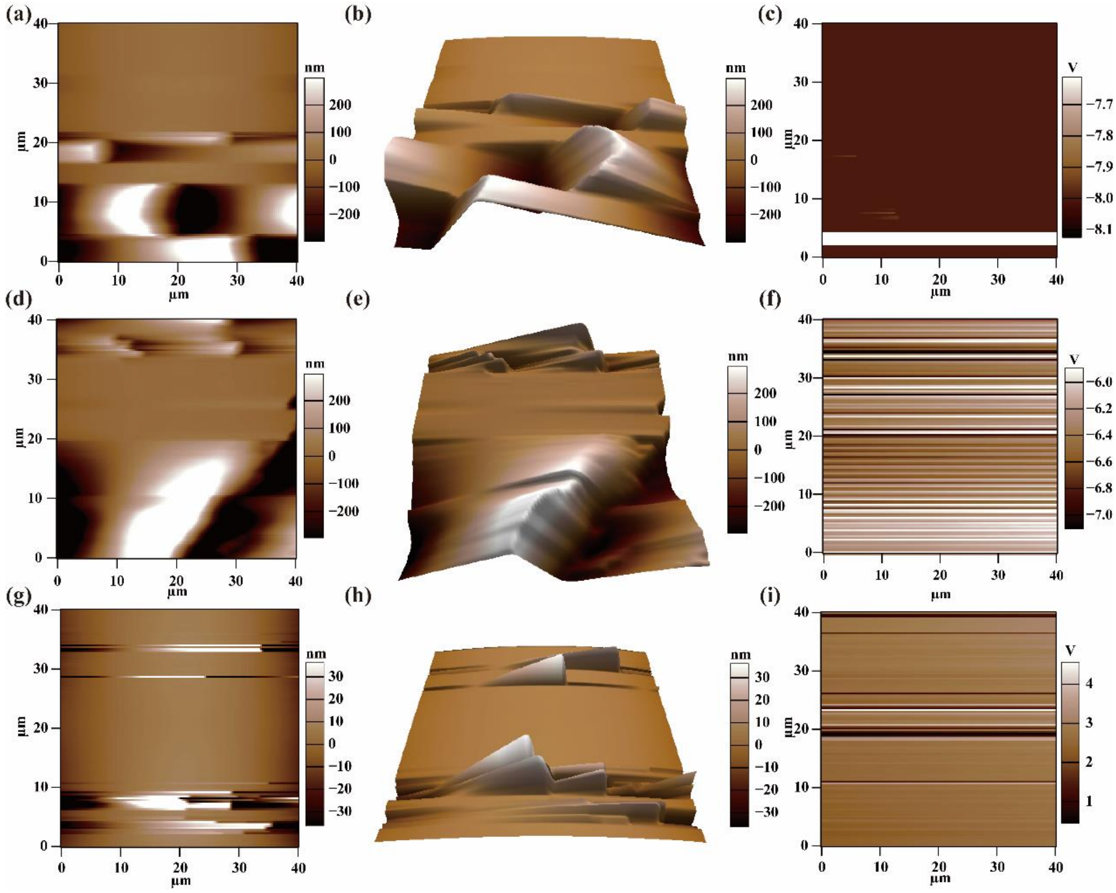

Figure S6. Measurement results of surface roughness and surface charge potential of the triboelectric layer. (**a**–**c**) MXene-doped PDMS: (**a**) top view of surface roughness, (**b**) 3D view of surface roughness, and (**c**) KPFM image of MXene-doped PDMS. (d-f) Graphene-doped PDMS: (**d**) top view of surface roughness, (**e**) 3D view of surface roughness, and (**f**) KPFM image of Graphene-doped PDMS. (**g**–**i**) PA6: (g) top view of surface roughness, (**h**) 3D view of surface roughness, and (**i**) KPFM image of PA6.

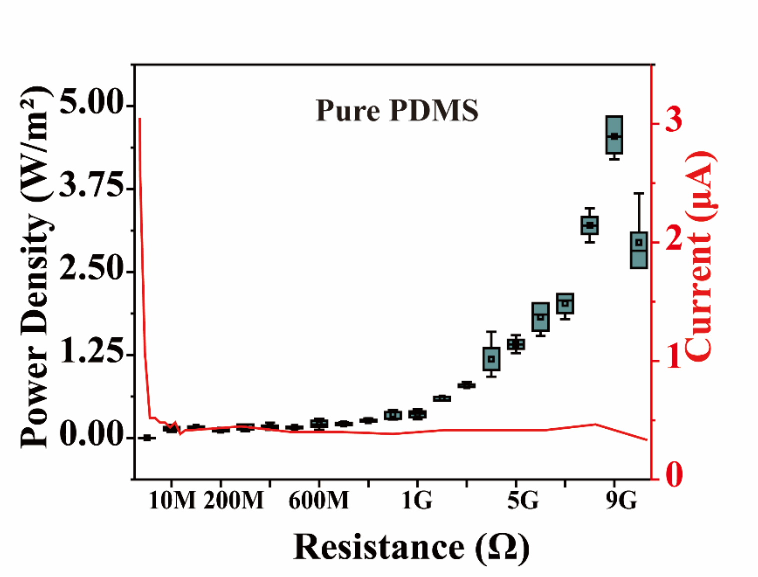

Figure S7. Power density and current curves under different load resistance for pure PDMS-based TENG.

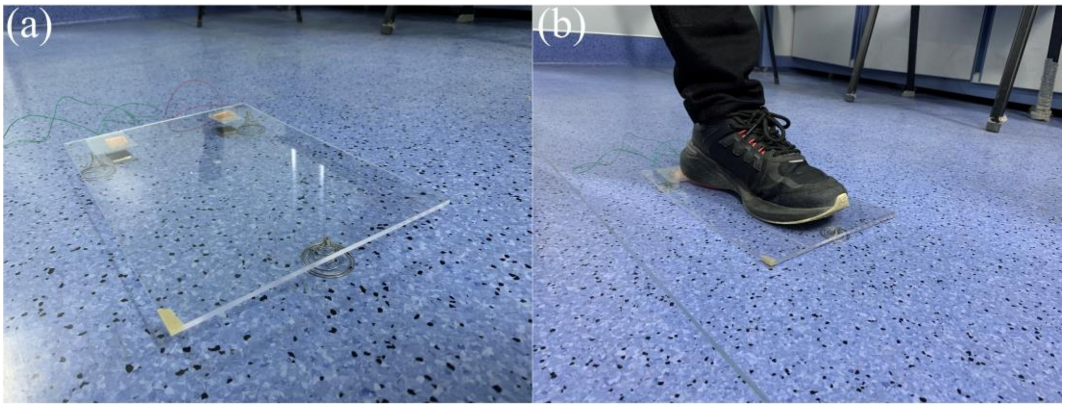

Figure S8. (**a**) Photograph of the health monitoring apparatus based on two separate MXene-doped LLB TENGs. (**b**) Photograph during the test.

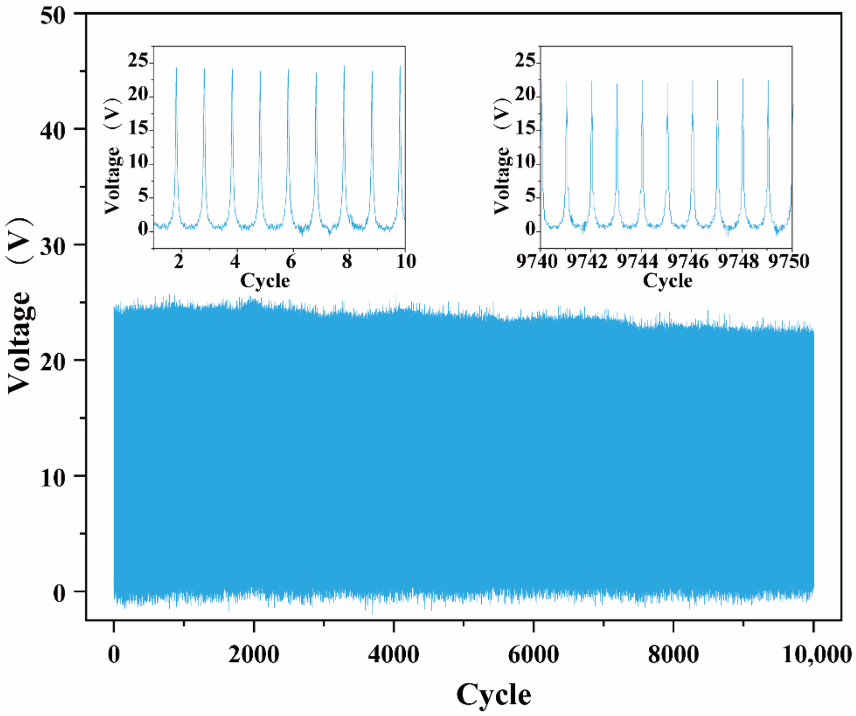

Figure S9. Reliability of the outer sensor under 10,000 working cycles, showing stability and repeatability of the device. The insets are the output voltage signal plots at the beginning and at the end stage of the test.

Video S1: Driving LED arrays.
Video S2-1: Driving electronic watch.
Video S2-2: Driving electronic watch.
Video S3: Feet sensor test.
